# Allies or enemies? The effect of regulatory T cells and related T lymphocytes on the profibrotic environment in bleomycin-injured lung mouse models

**DOI:** 10.1007/s10238-022-00945-7

**Published:** 2022-11-20

**Authors:** Mutlu Seyran, Scalise Melanie, Stumbles Philip, Gazdhar Amiq, Blank Fabian

**Affiliations:** 1 Department for BioMedical Research DBMR, Department of Pulmonary Medicine, Inselspital, Bern University Hospital, University of Bern, Bern, Switzerland; 2 Telethon Kids Institute, University of Western Australia, Nedlands, WA Australia

**Keywords:** Bleomycin injury, Mouse lung injury model, T cells, IPF, Lung fibrosis, Immunomodulatory effect

## Abstract

Idiopathic pulmonary fibrosis (IPF) is characterized by permanent scarring of lung tissue and declining lung function, and is an incurable disease with increase in prevalence over the past decade. The current consensus is that aberrant wound healing following repeated injuries to the pulmonary epithelium is the most probable cause of IPF, with various immune inflammatory pathways having been reported to impact disease pathogenesis. While the role of immune cells, specifically T lymphocytes and regulatory T cells (Treg), in IPF pathogenesis has been reported and discussed recently, the pathogenic or beneficial roles of these cells in inducing or preventing lung fibrosis is still debated. This lack of understanding could be due in part to the difficulty in obtaining diseased human lung tissue for research purposes. For this reason, many animal models have been developed over the years to attempt to mimic the main clinical hallmarks of IPF: among these, inducing lung injury in rodents with the anti-cancer agent bleomycin has now become the most commonly studied animal model of IPF. Pulmonary fibrosis is the major side effect when bleomycin is administered for cancer treatment in human patients, and a similar effect can be observed after intra-tracheal administration of bleomycin to rodents. Despite many pathophysiological pathways of lung fibrosis having been investigated in bleomycin-injured animal models, one central facet still remains controversial, namely the involvement of specific T lymphocyte subsets, and in particular Treg, in disease pathogenesis. This review aims to summarize the major findings and conclusions regarding the involvement of immune cells and their receptors in the pathogenesis of IPF, and to elaborate on important parallels between animal models and the human disease. A more detailed understanding of the role of Treg and other immune cell subsets in lung injury and fibrosis derived from animal models is a critical basis for translating this knowledge to the development of new immune-based therapies for the treatment of human IPF.

## Background

Idiopathic pulmonary fibrosis (IPF) is a lethal disease with an increase in prevalence over the past decades [1, 2]. The pathogenesis of the disease is based on aberrant wound healing mechanisms within the lung driven by repeated micro-injuries leading to scarring followed by fibrosis [3]. These processes will lead at later stages to dyspnea and a dramatic decrease in lung function. Even after decades of investigations, the exact etiology of lung fibrosis remains unknown. Thus, it is crucial to clearly determine and understand the pathophysiology leading to lung fibrosis. In order to investigate the pathogenesis of IPF, different animal models mimicking the characteristics of pulmonary fibrosis have been employed in order to dissect the mechanisms of the human disease [4]. The main pathological features displayed in these models are an excessive accumulation of extracellular matrix (ECM) within the lung, distortion of the lung architecture, immune cell infiltration and decrease in lung function [5]. The best-described animal model of IPF is based on the administration of bleomycin, an anti-cancer agent known to cause lung fibrosis in patients undergoing chemotherapy to treat tumors [6]. Therefore, bleomycin administration is a method of choice to induce lung fibrosis in experimental animal models. Intra-tracheal administration of bleomycin directly to the lung first causes a phase of inflammation, which leads to fibrosis after few days (the timing depends on the animal strain), and many characteristics shared with human lung fibrosis may be observed, including septal thickening and increased collagen deposition within the lung alveoli [5].

**Fig. 1:**
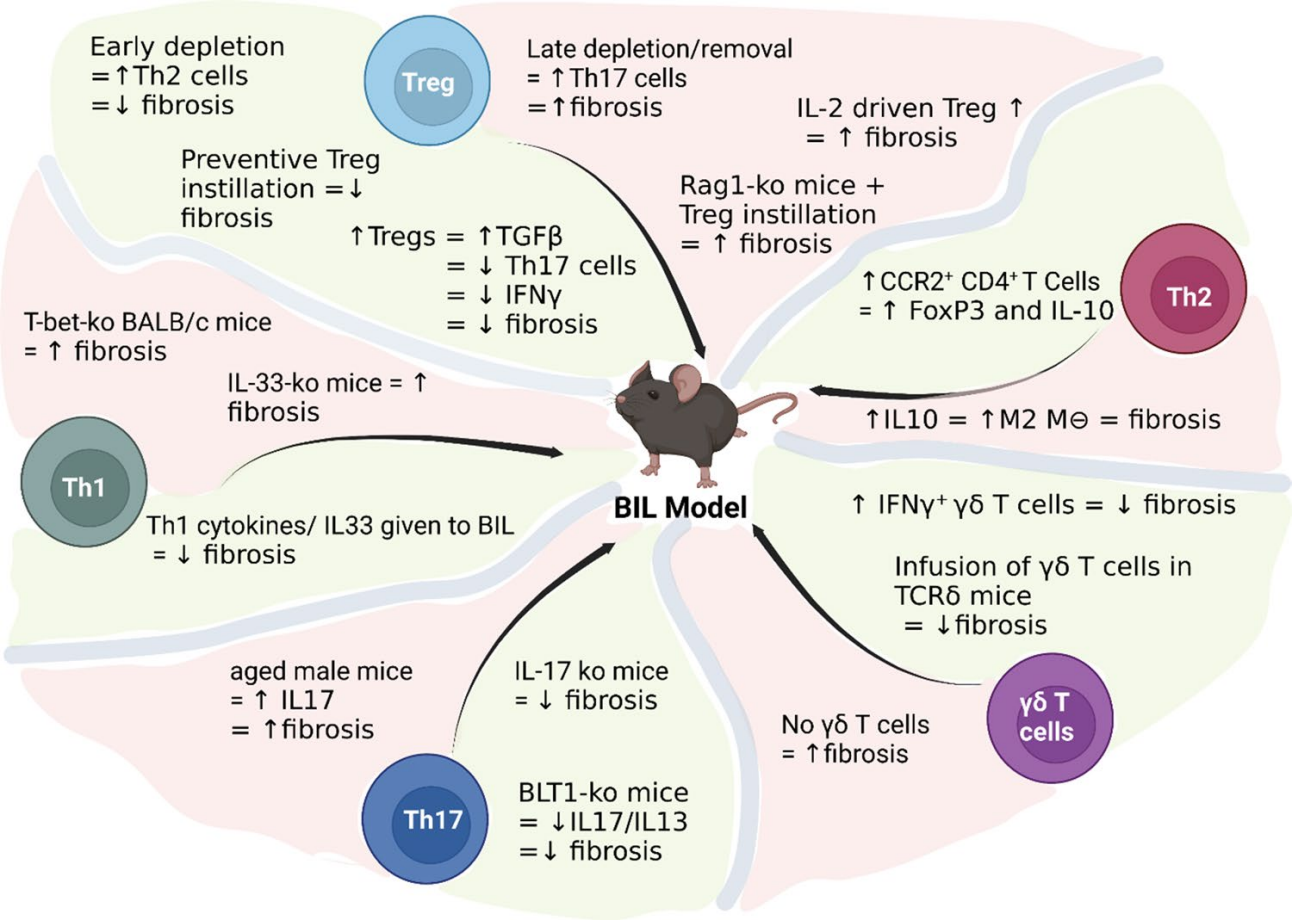
Implications of local effector T cell subsets implications on the bleomycin-injured mice lung (BIL) model. Beneficial and adverse impacts are highlighted in green and red, respectively. Figure created on *BioRender.com*

Although bleomycin-induced lung fibrosis in rodents is transient and not as chronic as in the human disease, many insights into human lung fibrosis have been obtained employing this model. Additionally, other methods to promote lung fibrosis in animal models have been used such as high dose irradiation, liposaccharide administration and other procedures [7–9]. Lung fibrosis in human IPF and in animal models shares a dysregulated immune response, which is one of the main drivers of the disease. One important component of human disease is the lymphocytic compartment [10]: while initially B cells were thought to have less impact on the disease, more recent evidence suggests their essential role in the pathogenesis of the human disease [11, 12]. Additionally, T cells also appear to have a significant, although controversial, impact on the course of the human disease. However, evidence from the animal model suggests that the whole T cell compartment plays an important role in the establishment of fibrosis after initial inflammatory processes induced by bleomycin injury [13]. This is supported by evidence that activated T cells have been shown to be concentrated in interstitial fibrotic areas in the human fibrotic lungs [14, 15]. However, it seems that the potential beneficial or adverse effect that T cells could have during pulmonary fibrosis mainly depends on the observed T cell subset [16], an observation that also applies to animal models, where the different effector and regulatory T cell subsets seem to act differently during the course of the disease. However, it is important to consider different T cell subsets as acting both independently from each other and interconnected, depending on the tissue micro-environment and stage of disease. In this review, we will focus on the roles of different T cell subsets in the bleomycin-injured mouse lung model with an emphasis on regulatory T cells (Treg), discussing the controversial issues surrounding the role of Treg in the pathogenesis of lung fibrosis depending on the stage of the disease (Fig. 1). While we will focus in this review on the bleomycin-injured mouse lung (BIL) model, given that it serves as the most commonly used model for investigation by the vast majority of research teams studying lung fibrosis, evidence from other mouse models will also be discussed. As different studies have come to drastically opposing conclusions on the role of T cells and regulatory T cells (Treg) in lung fibrosis, in this review we will first describe what is known of the pathogenic role of specific T cell subsets in the animal model of bleomycin-induced lung injury before comparing this with what is known of their role in the pathogenesis of the human disease.

## The regulatory immune system in the aging lung

Being a disease afflicting individuals over 65 years of age, it is important to understand the impact of the aging processes on the pulmonary immune system and lung fibrosis before going in depth into the regulatory immune compartment and Treg in the bleomycin-injured mouse lung model, which will be further discussed below. Besides the morphological and functional changes occurring in the aging lung, the pulmonary immune system is aging as well and undergoes a similar decline in functionality and steadiness. In the aging lung, even without a pathological threat, there is a basal activation of the innate immune system, which is not observed in younger lungs [17], a mechanism is referred to as “inflamm-aging”. Elevated levels of pro-inflammatory cytokines such as interleukin-(IL)1β, IL6 and tumor necrosis factor α (TNFα) were shown to be increased in aged lung [18–20]. The continued secretion of pro-inflammatory cytokines may cause a deterioration of the lung parenchyma over time [21]. Another phenomenon accompanying the inflamm-aging processes is immune-senescence, where the immune response appears to be suboptimal [22]. Several studies have shown that innate immunity seems to be impaired in the aged lungs, as well as its intrinsic link to the adaptive immune system [23, 24]. Indeed, in order to induce a powerful T and B cell response to a specific pathogen, CD4^+^ T helper cells need to be primed by dendritic cells (DCs) in the lung draining lymph nodes in order to become effector cells. In the aged lung, DCs have been shown to develop reduced homing capacity to lymph nodes probably linked to an observed decreased level of the chemokine ligand 21 (CCL21), which attract DCs to the lymph nodes through binding to chemokine receptor 7 (CCR7) that is present on their cell membrane [24]. Additionally, naïve CD4^+^ and CD8^+^ T cell numbers were shown to be reduced in both aged humans and rodents in comparison with memory T cells [25]. Also, the proliferative state of CD4^+^ T cells was reduced in the elderly after anti-CD3 treatment in comparison with the younger control group, which could coincide with a poor immune response to pathogens [26]. The constant inflammation observed in the inflamm-aging process may be worsened by a defective regulatory immune system due to Treg exhaustion in older subjects [27]. In addition, increased proportions of Treg were also observed in older human subjects in comparison with younger individuals [28, 29]. However, it is not certain if the increased percentage of Treg is due to a decrease in regular CD4^+^ T cells at older age or if certain subsets of Treg are expanding with the aging process [30, 31]. Furthermore, it was shown in a model of aged mice suffering from influenza virus-induced pneumonia that Treg were contributing to a dysfunctional immune response by inducing an impaired recovery after a viral attack on the lungs [32]. The number and functionality of specific Treg subsets appear to undergo dramatic changes in different age-related diseases such as cancer, neurodegenerative, cardiac and lung disorders, pointing toward the importance of these cells when considering Treg cell-based therapies [33].

### The role of T regulatory cells in fibrosis

In the broadest sense, T regulatory cells (Treg) are known to be immune response regulators, as they dampen immune mechanisms to promote organ homeostasis and peripheral tolerance [34]. More generally, Treg are known to be CD4^+^CD25^+/−^ Forkhead box P3^+^ (FoxP3) T cells [35, 36] and they express anti-inflammatory cytokines such as interleukin-10 (IL-10) or transforming growth factor β (TGF-β) or act directly on different metabolic pathways like cyclic AMP [37–39] (cAMP).

These cells appear to accumulate in different murine fibrotic models, including liver fibrosis, cystic fibrosis and cardiac fibrosis, and appear to undergo changes in expression of cell surface markers [40]. A study of IPF patients showed a global impairment of Treg, which correlated with increase in severity of the disease [41]. A similar observation was reported for liver fibrosis, where the balance of Treg over other type of lymphocyte changed the disease outcome [42]. Therefore, it is evident that Treg play a role in different fibrotic diseases in humans in addition to pulmonary fibrosis.

### The role of regulatory T cells in the bleomycin-injured mouse lung model

Different studies focusing on immune related receptors and cytokines have shown a positive impact on both bleomycin-induced fibrosis and Treg populations within the lung of bleomycin challenged mice. One study showed that fibrosis markers decreased in a CCR7-deficient mouse model after bleomycin challenge compared to wild-type mice challenged with bleomycin [43]. C–C chemokine receptor 7 (CCR7) is a receptor expressed on different immune cell types, whose ligands are CCL19 and CCL21 (C–C chemokine ligand 19 and 21), known lymphocytic attractants in secondary lymphoid organs [44]. Also, CCR7 is present in fibroblastic foci of IPF patients [45]. In the CCR7-deficient mouse model three weeks after bleomycin assault, a clear population of Treg (CD4^+^CD25^hi^FoxP3^+^) was observed in the lung, which was not present in wild-type mice upon same treatment. Two genes, interleukin-2 (IL-2) and indoleamine 2,3-dioxygenase (IDO), promoting a Treg favorable environment, were upregulated in CCR7-deficient mice. The authors suggested that the absence of CCR7 and the following upregulation of those two genes allowed retention of the Treg within the lung tissues after bleomycin administration. Recently, Takei et al. [46] focused on the effect of another receptor molecule connected to pulmonary fibrosis, called aryl hydrocarbon receptor (AhR). This receptor was over-expressed among immune system compartments [47] and proved to have modulatory effects, such as equilibrating T helper cells type 17 (Th17) versus Treg cell immune responses [48]. As a part of their study, the authors injected a ligand of the AhR, called FICZ [49] into mice following bleomycin administration. After FICZ administration, a decrease in collagen deposition was observed, which was concomitant with an increase in CD4^+^FoxP3^+^ Treg, and a decrease in interferon-gamma (IFNγ)^+^ CD4^+^ T cells and inflammatory γδ IL17^+^ T cells in lung homogenates compared to bleomycin instilled mice treated with vehicle (negative control treatment). They suggested that the variations in T cell subsets might be linked to an overall positive effect induced by FICZ administration following bleomycin injury in mice. Another study by Tang et al. [50] found that a mouse model overexpressing latent TGF-β_1_ in lung tissues showed dramatic reduction in different markers associated with pulmonary fibrosis and inflammation after bleomycin administration. TGF-β_1_ is a cytokine inducing FoxP3 expression and implicated in the upregulation of Treg response [51]. In the mouse model overexpressing latent TGF-β_1_ in lung tissues, they could observe an inactivation of the TGFβ/Smad3 and nuclear factor-kappa B (NF-kB) signaling pathways, which was concomitant with an increased proportion of CD4^+^FoxP3^+^ Treg in comparison with bleomycin challenged mice. This effect was accompanied by an increase in IL-10 mRNA expression within the lung. The latent TGF-β_1_ expression found in this mouse model seems to act on the balance between Th17 and Treg cells via downregulation of RORγt/IL17 and upregulation of FoxP3/IL-10. A recent study showed some recovery processes in an acute lung injury animal model, where the balance was shifted toward Treg response using the effect of TGF-β_1_ to promote a Treg prone environment [52]. It is important to mention that overexpression of latent TGF-β_1_ in lung tissues correlated with higher FoxP3 expression, without further activation of the TGF-β pathway. This mechanism could be one explanation why the mouse model overexpressing latent TGF-β_1_ developed less fibrotic lesions after bleomycin treatment. Taken together, these studies show that an increased Treg population is often linked with decrease in fibrosis in the bleomycin-injured mouse lung injury model.

On the other hand, in two interesting studies, Treg were depleted using anti-CD25 antibody at different time points of bleomycin administration in mice, to dissect the role of this T cell subset in development of fibrosis. Boveda-Ruiz et al. [53] observed that an early depletion of CD4^+^CD25^+^ Treg could prevent bleomycin-induced lung injury, associated with a predominance of T helper cells type 2 (Th2). However, late depletion of Treg worsened the inflammatory response and pulmonary collagen accumulation in the mice, this time associated with an increase in Th17 cells. The authors also observed an important increase in Treg in a humanized model of TGFβ_1_ transgenic mice, expressing human TGF-β_1_ directly in the lung, and concluded that predominance of specific subsets of T cells may depend on the inflammatory or fibrotic stages created by bleomycin administration. Chakraborty et al. [54] showed that the preventive Treg depletion before bleomycin administration restricted the collagen deposition within the lungs, which decreased the Ashcroft score in comparison with the bleomycin-injured control group. In addition, the depletion resulted in a decrease in Th17 cells and associated cytokines within the lung. This T cell subset is known to play a key role in bleomycin-induced lung fibrosis in particular in the murine model [52]. Another interesting effect was the increase in the cytokine IFNγ after depletion of Treg, suggesting a role for those cells in dampening of IFNγ release after bleomycin challenge. Both studies above suggested an overall beneficial effect after a preventive or early Treg depletion in bleomycin challenged mice, affecting other cell types and related cytokines. Another study investigated the effect of increased Treg proliferation. Birjandi et al. based their approach on the enhancement of in vivo CD4^+^CD25^hi^FoxP3^+^ Treg via IL-2 complex injection in mice [55]. They demonstrated that an increase in pulmonary Treg, induced by IL-2 complex administration, is associated with worsened lung fibrosis, after bleomycin injury shown by more collagen deposition. This increase in pulmonary Treg also shifted the immune environment toward a profibrotic Th2 immune response. Furthermore, this study showed that Treg administrated to recombinant activating gene 1 (Rag1) deficient mice, lacking T and B cells, enhanced mortality in mice and was linked to a dramatic increase in profibrotic processes within the lungs after bleomycin administration. They suggested that this type of Treg underwent some phenotypical switch upon bleomycin challenge, which promoted their profibrotic action. Overall, based on these studies, we may conclude that promotion of Treg in an early stage of bleomycin effect will enhance the associated fibrotic markers. However, the depletion of Treg at later stages may induce adverse profibrotic effects. Finally, the dysregulation of Treg has a dramatic incidence on some other cell types as Th17 for example.

### Treg cell therapy

In this section, we will highlight the potential of Treg related therapies as possible novel treatment approaches for fibrosis. An interesting approach given by Tan et al. [56] showed the unique ability of human amnion epithelial cells (hAECs) to promote CD45^+^FoxP3^+^ Treg proliferation and their pulmonary recruitment after bleomycin challenge in the mice. The administration of both hAECs (intraperitoneal) and Treg (intravenous) after bleomycin challenge in a Rag1-deficient mouse model mitigated the fibrotic process usually induced by bleomycin administration. Interestingly, only Treg or concomitant non-Treg and hAECs adoptive transfer failed to prevent the development of fibrotic lesions. A research group tried to reverse the fibrosis caused by bleomycin in mice via caudal vein injection of splenocytes [57]. Administration of splenocytes promoted the recovery from fibrosis in mice. However, under same treatment, Treg depletion using anti-CD25 antibody resulted in abrogation of the amelioration, even increasing fibrotic readouts. Based on this observation, they suggested an immunomodulatory effect of Treg on other cells. Following this conclusion, they adoptively transferred Treg in the bleomycin challenged mice. A significant attenuation of fibrosis, demonstrated by a decrease in lung hydroxyproline content, could be observed following Treg administration. Moreover, they also demonstrated that splenectomy before bleomycin administration reduced fibrosis within the lungs. Recently, Zhang et al. [58] investigated the effect of CD4^+^CD25^+^ Treg on the Th1/Th2 balance; the authors observed variations in Treg proportions three weeks after bleomycin instillation. An increase in Treg during the first days was observed, followed by a decrease in the Treg population within blood and spleen until 14 days, and finally a smooth increase at 21 days after administration. They performed Treg adoptive transfer on two different time points. A preventive transfer, four days before bleomycin instillation and an acute transfer, where cells were injected four days after bleomycin instillation. In the first observation, even if fibrosis was present on days 7, 14 and 21 after bleomycin administration, the preventive transfer of Treg markedly decreased progress of fibrosis compared to bleomycin administration alone. This decrease in fibrosis was demonstrated by reduced pulmonary hydroxyproline content. The authors proposed that injection of Treg before bleomycin administration augmented the amount of exogenic Treg present in the lung, which could have triggered a more tolerogenic environment thus preventing a worsened fibrosis. Second, the acute adoptive transfer of Treg showed a higher inflammatory response and seemed to worsen the effect of bleomycin in the lungs of treated mice. In summary, the authors suggested that the proportion of Treg in the peripheral blood could follow the sequence of the different phases of pulmonary fibrosis at least in mice. It may be interesting to check the correlation of the Treg level in the blood with the stage of pulmonary fibrosis in humans. Those studies seem to point toward a time-dependent ameliorating effect of Treg on the course of fibrosis. Removing Treg from the environment as well as an infusion of Treg after bleomycin assault seems to impair the course of the disease. However, infusing Treg in Treg depleted mice or preventive infusion of exogenic Treg before bleomycin injection seems to ameliorate the disease. Taken together, these results showed a critical time-dependent utilization of Treg as therapeutic agent that should be precisely determined prior to any treatment. With a time-dependent manner of Treg delivery, decreased fibrotic markers could be observed and on the contrary, without this tightly regulated schedule the effect could be worsened.

### The role of other T cell subsets in immune regulation and fibrosis

Here, we summarize the knowledge of other types of T cells or cytokines that have been implicated during bleomycin-induced lung injury and that have an impact on the Treg environment:

#### γδ T cells as notable promoters of a T regulatory environment

While it has been suggested that bleomycin-driven pulmonary fibrosis is promoted through a Th2-biased environment rather than a Th1 environment [59], some studies have emphasized an imbalance between Treg and Th17 cells as one important issue driving the fibrotic milieu [60, 61]. In this section, we will discuss the evidence showing the role of other subsets of T cells that could influence the Treg/Th17 balance and how they could be beneficial in the bleomycin-injured mouse lung model. For example, IPF patients have been shown to have decreased γδ T cell levels in bronchoalveolar lavage fluid (BALF) samples [62] while Segawa et al. showed that γδ T cells demonstrated an immune-regulatory role in the bleomycin-injured mouse model [63]. They based their approach on a T cell receptor (TCR)δ-deficient mouse model, in which bleomycin potency was increased when inducing pulmonary fibrosis. This was demonstrated with higher expression of TGF-β and collagen 1α1 (Col1α1) after bleomycin administration in comparison with controls. They also found that the proportion of IFNγ^+^ CD4^+^ T cells decreased and the IL17^+^ CD4^+^ T cell population increased in γδ T cell-depleted mice in comparison with controls. Administration of γδ T cells in the TCRδ deficient mice reduced this bias considerably. Moreover, using an IL-17-deficient mouse model, reduced pulmonary fibrosis after bleomycin assault was observed, as shown by less collagen deposition in comparison with control animals. In addition, in vitro co-culture experiments suggested that γδ T cells abrogated Th17 cell differentiation through secretion of IFNγ. Finally, to confirm the mode of action of γδ T cells to decrease bleomycin-induced pulmonary fibrosis, they infused IFNγ^+^ γδ T cells into IFNγ-deficient mice. This transfer of IFNγ^+^ γδ T cells significantly reduced collagen deposition within the lungs. Intriguingly, another study showed that IFNγ-deficient mice suffered less after bleomycin assault in comparison with controls [64]. These findings suggest a further beneficial role of IFNγ^+^ γδ T cells as a source of IFNγ^+^ in this animal model. Other studies showed that γδ T cell deficient mice presented an aggravated fibrotic response upon bleomycin challenge. In a study by Braun and colleagues, it was shown that lack of γδ T cells in mice increased collagen deposition and inflammatory makers, and delayed epithelial regeneration upon bleomycin challenge in comparison with wild type mice.

Interleukin-17 is another important mediator with potential effect on the course of pulmonary fibrosis. It is a pro-inflammatory cytokine produced mainly by CD4^+^ Th17 cells in humans and secreted by γδ T cells in mice [65]. It was shown that γδ T cells secrete IL17 upon bleomycin challenge and localize in the lesion caused by the bleomycin in the lungs [66]. The same study demonstrated the crucial role of γδ T cells in regulating the fibrotic pulmonary environment by being the major source of IL-17 during bleomycin-driven pulmonary fibrosis [66]. Another study showed that upon adoptive transfer of γδ T cells in γδ T cells-deficient mice, bleomycin-induced fibrosis was drastically reduced through the induction of CXCL10 secretion [67]. In summary, the studies above suggest that γδ T cells could play a role in counteracting bleomycin-induced lung injury in the bleomycin mouse model and that IL-17 levels correlate with the severity of fibrosis in this model.

#### A Th17 imbalance

Following the idea of a bias toward a pro-IL-17 environment after bleomycin administration in rodents, Redente et al. [68] introduced the interesting concept that hormone levels, in conjunction with the dose of bleomycin, may act as a confounding factor in the induction of fibrosis after bleomycin injury. They showed that males, and more specifically, aged male mice present more attributes linked with IPF than younger and/or female mice following bleomycin injury. Aged male mice also displayed an increased mortality in comparison with younger mice, and there was a clear association with an increased level of IL-17A in BALF of aged male mice with a higher level of fibrosis and cell infiltration present in the lung parenchyma. Although IL-17A has already been linked with IL-1β and induction of fibrosis after bleomycin assault [69], another immune receptor was found to have an impact on IL-17 response in a bleomycin mouse model. Lv et al. [70] based their research on a leukotriene B4 receptor 1 (BLT1) deficient mouse model challenged with bleomycin. BLT1 is one of the receptors of the potent activator and attractant of lymphocytes called leukotriene B4 (LTB4) [71] and is shown to have great effect in inflammatory mechanisms and therefore, used in clinics to decrease the process in some chronic and acute inflammatory disorders [72]. The authors showed that BLT1 deficiency in mice induced a reduction of IL13^+^ and IL17^+^ CD4^+^ T cells and attenuated pulmonary fibrosis in comparison with control [70]. First, they showed that CD4^+^ T cells may not be a main actor in the bleomycin mouse model, since CD4^+^ T cell-depleted mice showed a similar establishment of fibrosis after bleomycin administration as control mice. Second, they could show that CD4^+^ T cells were not the major source of IL-17A, as 70% of IL-17A^+^ cells were found to be CD4^−^ in their mouse model. They concluded that the lack of BLT1 decreased the fibrosis on one hand through reduction of the neutrophilic inflammation phase and on the other hand through a dramatic decrease in the total T cells during profibrotic phase. Taken together, these studies show a clear link between IL-17 concentration and the course of fibrosis in the BIL mouse model.

#### Other immune-regulatory-related receptor pathways

Milger et al. [73] demonstrated an increase in CCR2^+^CD4^+^ T cells with potential anti-inflammatory and anti-fibrotic effect in IPF BALF samples. CCL2 acts as a chemoattractant for lymphocytes, especially T cells, via its CCR2 receptor [74]. These cells upregulate FoxP3 and IL-10 following bleomycin treatment in comparison with controls. This is particularly interesting as both factors are widely associated with Treg as FoxP3 is the main Treg transcription factor [75, 76]. Furthermore, human IPF and non-IPF BALF samples showed a higher proportion of CD25^+^FoxP3^+^ Treg cells in CCR2^+^CD4^+^ T cells than in CCR2^−^ CD4^+^ T cells, pointing toward a regulatory role of CCR2^+^CD4^+^ T cells. Increase in CCR2^+^CD4^+^ T cells in murine fibrotic lungs is an analogue finding in human IPF, showing an increased level of its ligand CCL2 in BALF [77] and in the alveolar epithelium [78]. Furthermore, the authors adoptively transferred the same subset, two days after bleomycin instillation, and on day 12 followed by an amelioration of fibrosis in comparison with control mice. This amelioration was observed by less collagen deposition in the lung, overall better lung compliance and less inflammatory cells present in BALF.

A study conducted by Xu et al. showed that BALB/c Tbet-ko mice were more sensitive to bleomycin-induced lung injury than controls [79]. The T box transcription factor TBX21 (Tbet) is known to be an important transcription factor leading to Th1 immune response and IFNγ production [80, 81]. In the literature, Tbet-ko mice are described as lacking Th1 immune response and overexpressing Th2 cytokines [82], an imbalance in the immune response which may intensify bleomycin-induced fibrosis as discussed above. The study from Xu et al. is interesting, as wild-type BALB/c mice are relatively resistant to bleomycin-induced injury compared to other mouse strains, and do not develop pulmonary fibrosis after high dose administration. However, upon bleomycin challenge BALB/c Tbet-ko mice showed an increased IFNγ expression, mostly driven by CD8^+^ T cells, as well as increased IL-10 expression. IL-10 is known as an anti-inflammatory cytokine often found in the fibrotic milieu of BIL animal model produced by different type of immune cells, such as Treg. Intriguingly, a study showed that IL-10 overexpression in a bleomycin mouse model, a specific macrophage subset called alternatively activated M2 macrophages arise within the murine lungs, followed by enhanced collagen accumulation and induced fibrocyte recruitment [83]. Finally, in vivo depletion of CD4^+^ T cells in Tbet deficient mice decreased both bleomycin-induced lung injury and expression of TGFβ within the tissue.

#### Cytokines/chemokines

IL-33 is an alarmin implicated in many inflammatory diseases and its receptor ST2 was identified as potent biomarker for heart failure and myocardial infarction [84]. Mature IL-33 was shown, in bleomycin-driven pulmonary fibrosis, to promote a Th2 cytokine profibrotic environment [85]. Liu et al. showed that IL-33 may dampen early inflammatory response, by stimulating Treg to release IL-13, and preventing mortality in a bleomycin mouse model [86]. They demonstrated with an IL-33 deficient bleomycin mouse model, enhanced levels of fibrosis related markers and increased mortality compared to controls. Further, they treated IL-33-deficient bleomycin challenged mice with rIL-33, which promoted ST2^+^Treg proliferation. ST2 is the receptor of IL-33, expressed by different cell types, including Treg [87]. The promotion of ST2^+^Treg was shown to reduce fibrosis induced by bleomycin treatment, which was suggested to be driven by IL-13 released by Treg. Indeed, after rIL-13 administration to IL-33 deficient mice, mortality level decreased after bleomycin challenge. A study by Luzina et al. showed high expression level of IL-33 in the respiratory tract of human IPF patients and in bleomycin-injured lung animals [88]. In the bleomycin-injured mouse lung model, both bleomycin and IL-33 were independently provoking fibrosis when administered to the lungs. However, the effect was more dramatic when both were administered together. The authors observed more collagen deposition after the dual administration than with either bleomycin or IL-33 alone. Thus, similar to a Th1 cytokine administered to mice after bleomycin challenge, IL-33 reduced fibrotic impact on the lungs. The authors suggested that this effect may be mediated partly through serum reduction of the cytokines TNFα [89, 90], IL6 [91, 92] and TGFβ [93], known to be involved in the fibrotic process of animal models and human IPF [88]. From the above, we conclude that IL-33 has an effect on the Treg environment, which may further drive fibrosis in bleomycin challenged mice.

## Discussion

In this review, we have discussed the bleomycin-injured mouse lung model based on the particular aspects of T cells and Treg cells related to their regulatory environment and functions that have been evaluated in a number of individual studies. It is very important that specific findings have to be compared very cautiously in order to draw a final conclusion on potential immunomodulatory mechanisms over the course of this disease. Every research laboratory has its own point of view based on their focus of research and on the model establishment and handling, dose and route of bleomycin administration and the final end point of experiments. In analyzing the existing literature, it has been very challenging to draw a clear map on specific T cell-driven mechanisms and effects in the bleomycin-injured mouse lung model and to evaluate possible parallels with its human counterpart, IPF.

One great issue lies with the lack of standardization and with the use of protocols to induce bleomycin-driven lung injury. The problem starts with ambiguous declaration of employed bleomycin dosage. Some protocols describe bleomycin in units per µl (U/µl) while some methods use µg/µl for the administration to the animal, which may render it very difficult to accurately reproduce specific conditions in the BIL model. It is important to mention that bleomycin concentration is known to have a considerable effect on the establishment of fibrosis in animal models [94], not to mention the species-dependent and the strain-dependent differences, which have to be carefully taken into consideration when choosing a specific bleomycin animal model. This point seems obvious as each strain shows a different susceptibility to the drug [95] and may be complicated even more so in the light of varying potency of bleomycin dependent on different companies and lot numbers. However, even with the same strain employed, bleomycin dosage may double from one study to another, rendering comparison, interpretation and reproducibility very difficult. However, for some studies, pilot experiments have been performed with different concentrations of bleomycin employed, in order to achieve optimal dosage for their specific model [96]. Unfortunately, most studies do not explain why they chose a certain dose of bleomycin. In a similar context, it is well known that human IPF is age and gender dependent, where the majority of patients are male and over 60 years old [2, 97]. This important fact is rarely taken into consideration when deciding on a specific animal experimentation design. Almost no research groups work with aged animals, except for very few groups that have just recently used aged mice to study lung fibrosis [68, 98, 99]. Furthermore, there are still many research groups working with female mice instead of males, even if the female hormonal cycle demonstrated a clear impact on the course of pulmonary fibrosis in animal and human IPF [100, 101]. Those considerations render an inter-study comparison very difficult, not to mention the challenge when trying to extrapolate specific findings to human IPF. Another problem emerges when looking for the “ideal” time point after administration in order to obtain the most representative readouts. Quite often studies desist from employing a time course in order to evaluate the course of the disease model and to collect important data on the dynamics of specific mechanisms. Readouts may change from “neglectable” to “massive” depending on the time point and the analysis was performed, as some papers have demonstrated the effect of bleomycin administration over period of time [102]. Monitoring the model at different time points allows improved accuracy when studying crucial effects on overly sensitive immune cells and in particular T cells and their subsets.

Identification and characterization of T cells is complex and requires multiple markers, the expression of which depends on various factors. Therefore, the use of markers used to delimitate specific cells for the Treg subset, as an example, further complicates the readouts. In some studies, the Treg subset is determined as “CD4^+^CD25^+^FoxP3^+^” [43, 55], while in others as “CD4^+^FoxP3^+^” [46, 50, 56] or “CD4^+^CD25^+”^ [53, 58] with or without adjunction of other markers. The comparison between those differently characterized Treg subsets should be done very prudently as we are not comparing identical subsets. It is quite the same scenario for the human IPF literature where flow cytometry analysis is done using completely different markers to define subsets of Treg [41, 103, 104]. Therefore, any conclusion may be drawn with care in the absence of standardized characterization of T cell subsets, as some studies may demonstrate adverse effects and others alleviating effects during the course of the disease with the “same” T cell subset, which may be different subsets in reality. Another problematic point is the use of anti-CD25 antibody as a Treg depletion mechanism. CD25, also known as IL-2R [105], is an immune receptor, which is not only expressed on Treg but on a variety of immune cells, including a subset of dendritic cells [106] and activated CD8^+^ T cells [107] in aged mice. A subset of CD25^+^ dendritic cells was shown to promote autoreactive Th17 cells in an autoimmune experimental uveitis model [108], when authors came to a similar conclusion as suggested by Boveda-Ruiz et al. [53] regarding Treg depletion acting on Th17 cells and their related cytokines. CD25 depletion will not only affect Treg, but all CD25^+^ subsets including their specific effect on Th17 cells.

As for other types of T cells, γδ T cells seem to have an overall beneficial effect against bleomycin assault, keeping in mind their versatility in expressing also profibrotic cytokines such as IL-6 and IL17 [109, 110]. It is interesting to mention that γδ T cells are known producers of CXCL1, IL6, IL17 and CXCL10 in mice, which seems contradictory as IL6 [111] and IL17 [109, 110] are known as profibrotic cytokines. From those studies, we can conclude that γδ T cells seem to secrete a wide variety of cytokines. Some of those have been shown to aggravate bleomycin-induced injury while some others, such as CXCL10 [67], showed protective effect. T cell-related cytokines and receptors such as IFNγ, CCR2 and BLT1 present alleviating effects on bleomycin in comparison with some others, such as IL1β and TGF-β, which further drive the fibrotic mechanism.

The cytokine IL-33 wears conflicting conclusions on the pulmonary fibrosis driven by bleomycin. Another key player we should have in mind, when talking about IL-33 and its receptor ST2, is the innate lymphoid cell type 2 (ILC2). ILC2 are innate immune cells capable of inducing a Th2 response upon IL-33, IL-25 or thymic stromal lymphopoietin (TSLP) [112]. Those cells are expressing the IL-33 receptor ST2, and IL-33 secretion was linked with an ILC2 promotion [113]. ILC2 presence in the lung tissue together with IL-25 secretion was showed to be increased in BALF of IPF patients [114]. It is interesting to mention that TGFβ and ILC2 were linked with pulmonary fibrosis pathogenesis in the murine model [115]. ILC2 concentration in blood correlated with poorer diagnosis in human IPF patients [116]. The profibrotic action of ILC2 cells was showed to be partially regulation by the RNAse, regnase-1 in the BIL model [117]. A study showed that Treg increase lead to a decrease in ILC2 cytokine secretion in a murine asthma model [118]. Moreover, TGF-β was showed to promote the expression of IL-33 receptor gene, ST2. Treg and ILC2 interplays show an interesting area of research that might be useful in understanding pathogenesis of the BIL model and at a clinical level.

How Treg and other regulatory immune cells are involved in anti- and profibrotic mechanisms in the lung is still not completely understood and remains of great interest. As one insight, it was demonstrated that myofibroblasts were susceptible to CD4^+^ T cell-driven apoptosis. In vitro*,* myofibroblasts from IPF patients were not susceptible to T cell-driven apoptosis and were secreting soluble Fas (sFas) ligand. Upon Fas-ligand addition in the culture, control myofibroblasts may acquire resistance to T cell-driven apoptosis [119]. It is interesting to mention that patients suffering from IPF demonstrated elevated level of circulating sFas death receptor ligand [120]. Treg may also promote fibrogenesis through TGF-β, IL-1 by inhibiting the recruitment of fibrocytes via the CXCL12/CXCR4 axis in a mouse model of acute lung injury [121]. It was also proposed that Treg may mitigate this recruitment via fibroblast growth factor 9 secretion [122]. Different studies show that Treg may lose their immunomodulatory effect during IPF, promoting a Th2-driven profibrotic environment [55, 123]. Further investigation regarding the inner mechanisms Treg are using to act on the fibrosis itself need to be addressed in order to have a better overview of their action mode in model of bleomycin-induced lung injury and in IPF itself.

## Conclusion

The literature regarding involvement of T cell subsets in the course of pulmonary fibrosis is quite dense, with many publications over the last decades touching on the implication of T cell subsets in human IPF or in animal models (Table 1). The task to delimitate clear conclusions from individual studies remains herculean when one looks closely at the plethora of non-standardized and incomplete reporting of methodological approaches, which affect the value of specific readouts in individual studies. As discussed in this review, the reported dosage to recreate the main features of pulmonary fibrosis varies greatly and inconsistent flow cytometry analysis follow quite the same trend throughout the literature. It is also crucial not to draw any conclusion from the whole T lymphocytic compartment and rather focus closely on well-characterized individual T cell subsets that may mediate adverse or beneficial effects in pulmonary fibrosis at least in the bleomycin-injured mouse lung model. In conclusion, there is an urgent need for accurate, standardized and uniform reporting in peer reviewed studies dealing with the interpretation of bleomycin-injured lung mouse model in particular. Reporting recommendations such as accurate description of the fibrotic substance used in the model (dose metrics!), the delivery vehicle, animal model and gender, exposure conditions to drug (if required), endpoints and statistics, etc., have to be established and strictly followed by each group. The impact of an individual study would benefit greatly from such a standardized approach, making the findings more relevant for comparison with endpoints from other studies following the same approach, including the validation, interpretation and cross-comparison of results.

**Table 1:** Different T cell subsets and their specific effect different mouse models of bleomycin-induced lung injury

T cell type	References	Mouse model + bleomycin-induced lung injury	Action on cells	Action on fibrosis	Other side effect(s)
**Treg action**	Trujillo et al. [43]	CCR7-ko mouse model	**↗** Treg	**↘** Fibrotic markers	IL-2 and IDO genes upregulation
	Takei et al. [46]	FICZ (ligand of aryl hydrocarbon receptor (AhR)), injected after bleomycin assault	**↘** IFNγ^+^CD4^+^ and inflammatory γδ IL17^+^ T cells **↗** Treg	**↘** Fibrotic markers	
	Tang et al. [50]	Overexpression of latent TGF-β	**↗** Treg	**↘** Fibrotic markers	Inactivation of the TGFβ/Smad3 and NF-kB signaling pathways **↗** IL-10 mRNA
	Wang et al. [52]	TGF-β injection after bleomycin assault	**↗** Treg	**↘** Fibrotic markers	FoxP3 expression ⍉ TGF-β pathway
	Boveda-Ruiz et al. [53]	Early Treg depletion using anti-CD25 antibody	⍉ Treg **↗** Th2 cells	**↘** Fibrotic markers	
		Late Treg depletion using anti-CD25 antibody	**↗** Th17 cells	**↗** Fibrotic markers	
		Humanized model of TGFβ1 transgenic mice	**↗** Treg		
	Chakraborty et al. [54]	Preventive Treg depletion	⍉ Treg	**↘** Fibrotic markers	
	Birjandi et al. [55]	IL-2 complex injection	**↗** Treg	**↗** Fibrotic markers	Th2 response
		Rag1 deficient mice injected with Treg	**↗** Treg	**↗** Fibrotic markers	
**Treg therapy**	Tan et al. [56]	Rag-1-ko mouse model + Treg injection + human amnion epithelial cells (hAECs)	**↗** Treg	**↘** Fibrotic markers	
		Rag-1-ko mouse model + Treg injection	**↗** Treg	**↗** Fibrotic markers	
		Rag-1-ko mouse model + non-Treg cells injection + human amnion epithelial cells (hAECs)	⍉ Treg	**↗** Fibrotic markers	
	Kamio et al. [57]	Caudal vein injection of splenocytes		**↘** Fibrosis	
		Caudal vein injection of splenocytes + Treg depletion using anti-CD25 antibody	⍉ Treg	**↗** Fibrosis	
		Treg instillation	**↗** Treg	**↘** Fibrotic markers	
		Splenectomy before bleomycin instillation		**↘** Fibrotic markers	
	Zhang et al. [58]	Preventive Treg instillation before bleomycin injury	**↗** Treg	**↘** Fibrotic markers	
		Treg instillation 4 days after bleomycin injury		**↗** Fibrotic markers	**↗** Inflammatory response
**Other T cells**	Segawa et al. [63]	TCRδ-ko mouse model, depleted of γδ T cells	**↘** IFNγ^+^ CD4^+^ T cells **↗** IL17^+^ CD4^+^ T cells	**↗** Fibrotic markers	**↗** TGF-β and COLIαI expression
		TCRδ-ko mouse model		**↘** Fibrotic markers	
		IL17-ko mouse model		**↘** Fibrotic markers	
		IFNγ-ko mouse model + instillation IFNγ^+^ γδ T cells		**↘** Fibrotic markers	
	Yamauchi et al. [64]	IFNγ-ko mouse model		**↘** Fibrotic markers	
	Lockhart et al. [65]	Lack of γδ T cells		**↗** fibrotic markers	
	Pociask et al. [67]	γδ T cells-ko mouse model + Adoptive transfer of γδ T cells		**↘** Fibrotic markers	
**Th17 cells**	Redente et al. [68]	Aged mice		**↗** fibrotic markers	**↗** IL17A level in BALF
	Lv et al. [70]	BLT1-ko mouse model	**↘** IL13^+^ and IL17^+^ CD4^+^ T cells	**↘** Fibrotic markers	
		CD4^+^ T cells-ko mouse model		= Fibrotic markers	
**Other**	Milger et al. [73]	BIL model	**↗** CCR2^+^CD4^+^ T cells		FoxP3 & IL-10 upregulation
		CCR2^+^CD4^+^ T cells adoptive transfer 2 days after bleomycin instillation		**↘** Fibrotic markers	
	Xu et al. [79]	Tbet-ko mouse model		**↗** Fibrotic markers	
	Sun et al. [83]	IL-10 overexpression	**↗** M2 macrophages	**↗** Fibrotic markers	
		Tbet-ko mouse model + CD4^+^ T cells depletion		**↘** Fibrotic markers	**↘** TGFβ expression
**Cytokines and chemokines**	Liu et al. [86]	IL-33-ko mouse model		**↗** Fibrotic markers	
		IL-33-ko mouse model + rIL-13 injection	**↗** ST2^+^ Treg	**↘** Fibrotic markers	

*Ko* Knockout, **↗** increase, **↘** decrease, ⍉ no/abrogation, BIL bleomycin-injured mouse lung model

## Data Availability

Not applicable.

## Abbreviations

AhR: Indoleamine 2,3-dioxygenase
BALF: Bronchoalveolar lavage fluid
BIL: Bleomycin-injured mouse lung model
BLT1: B4 receptor 1
cAMP: Cyclic AMP
CCL19,21: C–C chemokine ligand 19,21
CCR7: C–C chemokine receptor 7
Col1α1: Collagen 1 alpha 1
CXCL10: C–X–C motif chemokine ligand 10
ECM: Extracellular matrix
FoxP3: Forkhead box P3
hAECs: Human amnion epithelial cells
IDO: Indoleamine 2,3-dioxygenase
IL: Interleukin
IPF: Idiopathic pulmonary fibrosis
LTB4: Leukotriene B4
NF-kB: Nuclear factor-kappa B
RAG1: Recombination activating gene 1
Tbet: T box transcription factor TBX21
TCR: T cell receptor
TGFβ: Transforming growth factor beta
Th1,2,17: T helper cells type 1,2,17
TNFα: Tumor necrosis factor alpha
Treg: Regulatory T cells
